# Central coordination of electromotor and locomotor pathways in the weakly electric fish Brevimyrus niger

**DOI:** 10.21203/rs.3.rs-10782428/v1

**Published:** 2026-09-08

**Authors:** Maggie Stanford, Matasaburo Fukutomi, Caroline Haber, Bruce A. Carlson

**Affiliations:** Washington University in St. Louis; Hokkaido University; Washington University in St. Louis; Washington University in St. Louis

**Keywords:** optic tectum, sensorimotor integration, descending motor pathways, motor control, motor coordination, active sensing

## Abstract

Coordinating multiple motor outputs is essential for active sensing. Mormyrid weakly electric fish generate electric organ discharges (EODs) while swimming, providing a tractable system for studying central mechanisms of motor coordination. The optic tectum (OT), homolog of the mammalian superior colliculus (SC), is a conserved sensorimotor center that regulates orienting behaviors and is reciprocally connected with the central electromotor network in mormyrid fish. Further, stimulation of the OT can elicit fictive swimming. However, whether the OT coordinates locomotor and electromotor outputs remains unknown. By combining electrical recordings with precise behavioral tracking in freely swimming *Brevimyrus niger*, we found that changes in EOD rate reliably preceded swimming movements, suggesting a temporal hierarchy of motor control. In paralyzed *in vivo* preparations, local OT microstimulation elicited both fictive EODs and fictive swimming, with the same temporal ordering observed during behavior. Spatial analyses revealed that fictive swimming was elicited more by stimulation of deeper OT regions. Similarly, fictive EODs were elicited more by stimulation of deeper and more ventroposterior regions, consistent with previous anatomical findings. Furthermore, OT stimulation sites that elicited higher rates of fictive EODs also tended to elicit more fictive swimming, suggesting that the OT provides a common drive to electromotor and locomotor systems. Together, our results extend the conserved role of the OT/SC in coordinating orienting behaviors to the active electrosensory system of weakly electric fish.

## Introduction

During active sensing, animals coordinate multiple distinct motor outputs to efficiently process continuous streams of sensory information. For example, echolocating bats precisely coordinate sonar call production with aerial flight maneuvers and ear movements (Falk et al. 2015; Yin and Müller 2019). Likewise, rodents rhythmically coordinate breathing with whisking and exploratory head and nose movements (Berg and Kleinfeld 2003; Kurnikova et al. 2017). These studies together suggest that temporal coupling across motor systems is a fundamental feature of active sensing. However, little is known about the central mechanisms regulating such coordination.

Mormyrid weakly electric fish provide a unique opportunity to address this question. Mormyrid fish emit pulse-type electric organ discharges (EODs) while swimming to perform active electrolocation and communicate with conspecifics (von der Emde 1999; Hopkins 1999). EODs are generated by an electric organ located in the tail, whose excitable cells are derived from muscle tissue, and they create an electric field surrounding the fish. Specialized electroreceptors distributed across the skin detect perturbations in this field caused by nearby objects and EODs from conspecifics (Bennett 1971). Behavioral evidence suggests that EOD production may be co-regulated with swimming activities. For example, during agonistic encounters, fish increase their EOD rate while attacking an opponent (Bell et al. 1974; Kramer and Bauer 1976). During object approach, fish increase their EOD rate while decreasing their swimming speed (Hofmann et al. 2014, 2017). During prey capture, EOD accelerations accompany prey probing and size estimation (Arnegard and Carlson 2005). These findings indicate that EOD timing is dynamically adjusted according to behavioral context.

The neural networks controlling the temporal patterns of EODs have been well characterized. EODs are initiated by the command nucleus (CN) in the medulla, whose output is relayed via the medullary relay nucleus (MRN) to spinal electromotor neurons (EMNs) that innervate the excitable cells of the electric organ (Bell et al. 1983; Grant et al. 1986). The CN integrates inputs from the mesencephalic precommand nucleus (PCN), the thalamic dorsal posterior nucleus (DP), and the ventral portion of the toral ventroposterior nucleus (VPv) and thus serves as a final decision-making center for EOD generation (von der Emde et al. 2000; Carlson 2002a, 2003; Carlson and Hopkins 2004a). In addition, the CN sends an internal timing reference for EOD output through a corollary discharge pathway to the dorsal part of the ventroposterior nucleus (VPd), which in turn inhibits the DP and PCN, forming a recurrent neural network (Carlson 2002a, 2003; Carlson and Hopkins 2004a). Interestingly, the DP and VPd form reciprocal connections with the optic tectum (OT) (Wullimann and Northcutt 1990; Carlson 2002a).

The OT, homolog of the mammalian superior colliculus, plays a key role in sensorimotor integration (Isa et al. 2021). Its superficial layers primarily process visual input, while the intermediate and deep layers integrate multimodal sensory information and contribute to motor outputs (Basso and May 2017). The OT has been shown to be involved in the control of multiple types of movements in teleost fishes. For example, in goldfish, local electrical stimulation in the OT can evoke both eye and tail movements (Herrero et al. 1998). In larval zebrafish, optogenetic activation of OT neurons can evoke either ipsilateral (approach) or contralateral (escape) swimming by recruiting distinct downstream populations (Helmbrecht et al. 2018). In the mormyrid fish *Gnathonemus petersii*, local electrical stimulation of the OT can also evoke swimming activity, although its influence on EOD patterns has not yet been described (Requarth and Sawtell 2014).

In this study, we first performed simultaneous recording of EOD and swimming from freely behaving mormyrid fish, *Brevimyrus niger*, showing that the temporal pattern of EODs is correlated with tail bending. We therefore hypothesized that the OT may be involved in this temporal coordination. Using a paralyzed preparation, we systematically applied local electrical stimulation to specific regions of the OT and analyzed the resulting patterns of fictive EODs and fictive swimming. Our results provide a foundation for advancing the study of the neural basis underlying the temporal coordination of distinct motor activities during active sensing.

## Materials and Methods

### Animals

This study included a total of 28 *Brevimyrus niger* (behavior: n = 8; electrophysiology: n = 20) of both sexes ranging in mass from 4.3 g to 15.2 g and from 7.6 cm to 11.1 cm in total length. Fish were acquired from AliKhan Tropical Fish and were housed in water with a pH of 6–7, a temperature of 25–29°C, and a conductivity of 175–225 μS/cm. They were kept on a 12:12 light/dark cycle and fed frozen blood worms 4 times a week.

### Behavior

#### Experimental Apparatus

Simultaneous recordings of EODs and swimming activity were conducted in a circular tank made of polystyrene with a diameter of 28 cm. Individuals’ home tank water was used to fill the tank to a height of 8 cm. Swimming behavior was recorded using an infrared (IR)-sensitive video camera (See3CAM_20CUG, e-con Systems) positioned above the tank, under dark conditions with IR illumination. Two pairs of bipolar electrodes were placed orthogonally along the tank wall to reliably detect EODs from swimming fish, as the recorded amplitude of EODs varies with the fish’s orientation, and a single pair of bipolar electrodes may fail to capture signals when the fish is aligned perpendicularly. These electric signals were amplified 100x, bandpass filtered (10 Hz-20 kHz) (Model 3500, A-M systems), digitized at a rate of 48.8 kHz (RX8, Tucker-Davis Technologies), and stored using custom software based on OpenEx (Tucker-Davis Technologies). This software also sent 180 Hz trigger pulses to the camera shutter to synchronize the electric signal and video recordings.

#### EOD Analysis

EOD timing was extracted from two-channel electrical recordings using a threshold-based method. The timing of each EOD was defined as the first timepoint where the absolute value of either channels’ signal exceeded a threshold of 5 mV. To prevent multiple detections of a single EOD, we excluded events with inter-EOD intervals shorter than 1 ms. EOD rate was then computed using a spike density function by convolving each EOD time with a Gaussian kernel of 250 ms width.

#### Video Analysis

We used DeepLabCut to quantify the swimming movements of each fish from video frames (640 × 480 pixels) (Mathis et al. 2018). A total of 11 feature points were tracked: seven equally spaced points along the body axis from the snout to the tip of the tail, plus the base and tip of each pectoral fin.

Swimming speed was calculated from the x and y coordinates of the fish’s midpoint (fourth body axis point). Positional outliers were identified using a moving median-based method (window size: 30 frames) and replaced by linear interpolation. The resulting trajectory was then low-pass filtered using a third-order Butterworth filter (cutoff frequency: 10 Hz) to reduce high-frequency noise. Speed was computed as the frame-to-frame Euclidean distance between filtered positions, divided by the frame interval (~ 1/180 s).

Tail angle was calculated as the angle between two vectors consisting of the first and third body axis points (vector A) and the fifth and seventh body axis points (vector B). As with swimming speed, outliers in the tail angle series were identified using a moving median-based method and corrected by linear interpolation. The cleaned tail angle trace was then smoothed using the same third-order Butterworth filter.

#### Electrophysiology

Fish were anesthetized in 300 mg/L MS-222 and paralyzed via intramuscular injection of 0.1 mL of 3 mg/mL gallamine triethiodide (Flaxedil) (Sigma-Aldrich). Fish were transferred to a recording chamber filled with water where they were positioned on a plastic platform with a circular silver ground electrode surrounding the trunk, anterior to the fictive swim surgery site. They were submerged in freshwater except for the left dorsal musculature and head surface. Aside from where the surgery sites were located, any exposed skin was kept moist by covering the fish with damp Kimwipes. General anesthesia was maintained for surgery by respirating the fish with an aerated solution of 100 mg/L MS-222 through a pipette tip placed in the fish’s mouth. Fictive swim and OT surgery sites were anesthetized with 0.4% lidocaine applied to the skin. Individuals were head-fixed by removing a small segment of skin and underlying muscle near the OT surgery site to reveal the skull where a head-post was attached using a small amount of superglue.

#### Fictive Swim and OT Surgery

To expose the fictive swim recording site, scales were first removed from a small portion of skin dorsal to the lateral line and anterior to the dorsal fin. Using the myosepta as a guide, a segment of skin the width of three myomeres was removed to expose the musculature. To expose the OT, skin and muscle were first removed from the surgery site which was located slightly dorsal and posterior to the left eye. A segment of the skull and the left eye were removed to gain access to the entire surface of the OT and partially expose the telencephalon, cerebellum, and exterolateral nucleus (EL). The surrounding structures were used as landmarks in the calculation of the OT surface map.

After both surgeries were complete, the fish was brought out of anesthesia by switching to aerated freshwater respiration. After the completion of a recording session, the fish was switched back to anesthesia until fictive EOD production ceased. Individuals were sacrificed by freezing.

#### OT Map Calculation

Three landmarks were defined as landmark caudal (LC), landmark rostral (LR), and landmark ventral (LV) for the calculation of the OT surface map. LC was the halfway point along EL where it intersects with the dorsal-caudal OT, LR was the intersection of the cerebellum with the dorsal-rostral OT, and LV was the intersection of the telencephalon with the ventral-rostral OT. The x and y coordinates of each landmark were obtained from the micromanipulator (MP-285, Sutter Instrument company) holding the stimulus microelectrode. A 4 x 4 grid of the OT surface was calculated between the vectors LC-LR and LV-LR. Rows from dorsal to ventral were located at twenty percent increments of vector LV-LR and columns from rostral to caudal were located at twenty percent increments of vector LC-LR, each stimulus site being the location of an intersection. Stimulus depths of 50 μm, 200 μm, and 400 μm (lateral to medial) were calculated for each stimulus site from the z location of the stimulating microelectrode when touching the surface of the OT.

#### Stimulation of the OT

Electric stimuli were delivered via a concentric bipolar microelectrode (PTM3CC02INS, World Precisions Instruments) in either a 10-pulse stimulus train or a 500-pulse stimulus train consisting of 400 μs bipolar square pulses (100 μA, 100 Hz); parameters were controlled by an isolated pulse stimulator (Model 2100, A-M Systems). A total of either 50 or 30 trials were delivered to each OT stimulus site for individuals subjected to the 10-pulse stimulus. For those subjected to the 500-pulse stimulus, a total of 3 trials were delivered to each OT stimulus site.

#### Fictive Swim Recording

Recording electrodes for fictive swim (628000, A-M Systems) were pulled with a micropipette puller (Model P-97, Sutter Instrument Company), broken to a tip diameter of 50 μm, fire-polished, and filled with Hickman’s Ringer. The recording electrode was attached to the myoseptum between myomeres by applying negative pressure through a pipette holder (HL-U, Automatic Scientific) attached to a threaded syringe (728000, A-M Systems). A reference electrode consisting of silver wire was bent to a u-shape at one end and placed against the muscle as close to the recording electrode as possible. Fictive swim recordings were amplified 1,000x, band-pass filtered (100 Hz-1 kHz) (Model 1700, A-M Systems), digitized at a rate of 48.8 kHz (RX8, Tucker-Davis Technologies), and saved using custom written MATLAB software.

#### Fictive EOD Recording

To record fictive EODs (or EODCs) a pair of electrodes was oriented parallel to the electric organ (EO), held in place by a plastic tube. Fictive EOD recordings were amplified 1,000x, band-pass filtered (10 Hz-5 kHz) (Model 1700, A-M Systems), digitized at a rate of 48.8 kHz (RX8, Tucker-Davis Technologies), and saved using custom written MATLAB software.

#### Artifact Removal

Electrical stimulation of the OT resulted in stimulus artifacts present in both fictive EOD and fictive swim recordings. To remove artifacts, the values in each trace between the onset and offset of each stimulus pulse were interpolated(Heffer and Fallon 2008) using MATLAB’s linspace function. The onsets of stimulus artifact events were extracted from recordings of the stimulus trigger pulses. Using the sample points of stimulus onsets, the sample points of the stimulus offsets were determined using the duration of the stimulus pulse (400 μs) multiplied by the sampling rate (48.8 kHz). The values between onsets and offsets were replaced with values along a straight line calculated between the two points.

#### Determination of Fictive EOD and Fictive Swim Onset Timing

The onsets of fictive EODs were determined as the first negative peak in the three-peaked electromotor neuron potential (Bennett 1971; Carlson 2002a). To first decrease noise in fictive swim recordings, data were rectified and then smoothed using a Gaussian-weighted moving average filter with a window size of 1001 samples (~ 20.5 ms). The onset of each fictive swim bout was defined as the first time point at which the smoothed trace exceeded a threshold manually selected according to baseline noise level.

#### Calculation of Fictive EOD Rate and Fictive Swim AUC

Fictive EOD rate was calculated as the change in rate of fictive EOD production in response to electrical stimulation of the OT. The change in rate was calculated by subtracting the total number of fictive EODs in the pre-stimulus window (200 ms for the 10-pulse stimulus and 1000 ms for the 500-pulse stimulus) from the total number of fictive EODs in the stimulus window, both corrected for time. Values of Δ Fictive EOD rate are reported as fictive EODs per second (EODCs/s).

Fictive swim activity was calculated as the change in rectified, smoothed fictive swim activity in response to electrical stimulation of the OT. Values were determined by calculating the change in area under the curve (AUC). Pre-stimulus fictive swim activity was calculated as the sum of all points in the pre-stimulus window multiplied by the sampling interval (1/48.8 kHz). Fictive swim activity for the stimulus window was calculated in the same manner, and both were corrected for time. The change in fictive swim activity is the pre-stimulus activity subtracted from the peri-stimulus activity. Values of Δ Fictive Swim Activity are reported in nV.

#### Statistical Analysis

We analyzed data using MATLAB R2023b with the Statistics and Machine Learning Toolbox. Cross-correlation (xcorr) was used to examine the temporal relationship between EOD rate and swimming speed, and EOD rate and the absolute value of tail angle. Linear regression (fitlm) was used to evaluate the relationships between average fictive response onsets and fish length, between fictive EOD rate and fictive swim activity, and between fish length and average fictive EOD to fictive swim latency across individuals. To test the spatial dependence of fictive motor activities, we performed repeated-measures ANOVAs with depth (50 μm, 200 μm, and 400 μm), the dorsoventral axis, or the anteroposterior axis as the within-subject factor. For the latter two axes, positions 1–4 were coded as dummy variables. Prior to the ANOVA, the mean value for each plane was calculated for each individual. Finally, we fit a generalized linear mixed model (GLMM; fitglme) to confirm the relationship between fictive EOD rate and fictive swim activity while accounting for individual variation. Fish identity was included as a random intercept and EOD rate as a fixed predictor.

## Results

### Temporal coordination of electric signaling and swimming

We simultaneously recorded electric signals and swimming activity from freely swimming mormyrid fish in a circular tank (Fig. 1a–c). The fish exhibited shorter EOD intervals, or equivalently, higher EOD rates during swimming compared to resting periods (Fig. 1c). Cross-correlation analysis revealed a consistent temporal correlation between EOD rate and swimming speed across all eight individuals examined (Fig. 1d). A closer examination of the average cross-correlation function indicated that changes in EOD rate preceded changes in swimming speed by 40 ms on average (Fig. 1d). To further relate this relationship to physical dynamics of body movement, we measured the tail angle based on the posture of swimming fish (Fig. 1b). We found that the absolute value of tail angle also temporally correlated with EOD rate: changes in EOD rate preceded changes in tail angle by an average of 130 ms (Fig. 1e). Notably, fish occasionally produced transient increases in EOD rate (Serrier and Moller 1989; Carlson 2002b; Carlson and Hopkins 2004b), and some of these EOD bursts were temporally coupled with subsequent tail bending (Fig. 1f).

These behavioral analyses show that EOD production and swimming are temporally coordinated in this mormyrid fish. Our results suggest that this temporal coordination may be mediated by interactions between the separate motor pathways controlling EOD production and swimming.

### Timing of fictive EOD and fictive swim motor responses evoked by OT stimulation

Electrical stimulation of the deep OT (400 μm from surface) evoked both fictive EOD and fictive swim motor responses. In fish exposed to the 10-pulse stimulus, fictive swim typically occurred as one or two short bursts (Fig. 2). In contrast, the 500-pulse stimulus often evoked multiple bursts, indicative of sustained swimming (Fig. 3). Both stimulus types evoked robust increases in fictive EOD production. Among fish exposed to the 500-pulse stimulus, there was variation in the number and pattern of evoked fictive swim bursts: some fish exhibited rhythmic bursting throughout the entire stimulus (Fig. 3b) while others showed less frequent bursts separated by longer intervals (Fig. 3a).

To compare the timing of fictive EOD and fictive swim responses, we extracted the onsets of each response across trials (Fig. 3c). Raster plots revealed a consistent delay between the onset of increased EOD production and the onset of fictive swim bursting. Peri-stimulus time histograms (PSTHs) showed that the increase in EOD production and the onset of fictive swim bursting were time-locked to OT stimulation but occurred with different latencies (Fig. 3d). Increases in fictive EOD production consistently preceded the onset of fictive swim bursting, suggesting a temporal sequence between the two responses that aligns with our behavioral observations.

### Variation in fictive EOD and fictive swim response latencies across individuals

Using recordings from both stimulus paradigms (10- and 500-pulse stimuli) that included both fictive EOD production and distinct fictive swim bursts, we extracted the first onset times of each response to compare their latencies across individuals (Fig. 4a). Fictive swim responses showed greater variability in onset timing, with mean fictive swim onset occurring at 250.0 ± 14.8 ms across individuals, whereas mean fictive EOD onset occurred at 14.0 ± 0.9 ms.

To examine factors that may contribute to this variability, we compared the mean first onset times for each fish to body length (Fig. 4b). Linear regression revealed a significant negative correlation between body length and mean first fictive EOD onset (R^2^ = 0.251, t_16_ = −2.31, *p* < 0.05), as well as a near-significant positive correlation between fish length and mean fictive swim onset (R^2^ = 0.206, t_16_ = 2.04, *p* = 0.059). We next examined intraindividual variability in the latency from mean first fictive EOD onset to mean first fictive swim onset and compared this value to fish length (Fig. 4c). Linear regression revealed a near-significant positive correlation between body length and mean latency difference across individuals (R^2^ = 0.213, t_16_ = 2.08, *p* = 0.054), with mean latency increasing by 0.193 s for each 1-cm increase in body length. Together, these results suggest that larger fish tend to exhibit greater temporal separation between fictive EOD and swim responses. The increase in latency difference with body length may reflect differential scaling of neural or biomechanical processes underlying swim initiation and EOD production.

### Regional organization of fictive motor responses within the OT

Because the OT possesses regional variability in sensory processing and motor control, we investigated whether the location of OT activation influenced fictive EOD and fictive swim responses. Electrical stimulation was applied to 16 sites across the OT at depths of 50 μm, 200 μm, and 400 μm, progressing from lateral to medial (Fig. 5a). A control stimulus was first delivered above the OT surface to confirm that responses were evoked specifically by stimulation within the OT.

To visualize the spatial distribution of evoked fictive motor responses, we generated heatmaps showing the average change in fictive EOD rate and fictive swim activity across stimulation sites (Fig. 5b). Repeated measures ANOVA revealed significant effects of stimulus depth on the rate of fictive EOD production (F_1, 33_ = 41.0, *p* < 0.001) and the amount of fictive swim activity (F_1, 33_ = 14.2, *p* < 0.001). In addition, we found that the fictive EOD rate depended on both the dorsoventral axis (F_1,50_ = 8.5, *p* < 0.01) and the anteroposterior axis (F_1,50_ = 21.3, *p* < 0.001) whereas the fictive swim activity depended on neither the dorsoventral axis (F_1,50_ = 1.2, *p* = 0.288) nor the anteroposterior axis (F_1,50_ = 3.0, *p =* 0.091). To relate these response patterns to OT anatomy, we overlaid the stimulation sites and corresponding heatmap values onto schematic representations of OT histology (Fig. 5c–d).

These findings indicate that fictive EOD responses are spatially organized within the OT, varying with stimulation depth as well as along the dorsoventral and anteroposterior axes, whereas fictive swim responses are modulated by stimulation depth but do not show significant spatial dependence in other OT dimensions. The spatial specificity of fictive EOD responses suggests that the electromotor pathway may preferentially engage specific subregions or laminae within the OT, while fictive swim responses may arise from more distributed descending circuitry.

### Coordination of fictive motor responses during OT stimulation

To evaluate the relationship between fictive EOD production and fictive swim activity across OT stimulation sites, we generated scatter plots using the heatmap values from the 17 individuals included in the OT mapping experiments. We plotted the change in rate of fictive EOD production (EODCs/s) against the change in rate of fictive swim activity (nV) (Fig. 6). Individual linear regressions revealed significant positive correlations between the two fictive motor responses in 8 out of 17 fish (*p* < 0.05). To assess whether this relationship persisted when accounting for individual variability, we performed a generalized linear mixed model (GLMM) on all data points, which confirmed a significant positive association between fictive EOD rate and fictive swim activity (β = 2.22 ± 0.317, *p* < 0.001). When combining all data points across individuals, linear regression similarly revealed a significant positive relationship between changes in fictive EOD rate and fictive swim activity (R^2^ = 0.137, t_810_ = 11.3, *p* < 0.0001). These results suggest that OT activity is associated with coordinated modulation of fictive EOD rate and fictive swim activity, indicating that the OT may provide a common driving force influencing electromotor and locomotor output.

## Discussion

We showed that electric signaling and tail bending are temporally correlated in freely swimming *Brevimyrus niger* (Fig. 1). This coordination may be mediated by interactions among distinct motor pathways, with the OT representing a candidate brain region performing this coordination. We further found that local stimulation of the OT could induce both EODs and swimming, albeit with different latencies (Figs. 2–4). Spatial analyses revealed that OT subregions that elicited higher rates of EODs also tended to elicit more swimming (Fig. 6), whereas hotspots for EODs were more spatially restricted than those for swimming (Fig. 5). Together with prior anatomical evidence (Carlson 2002a), our results provide physiological support for the possibility that the OT is involved in the coordinated control of EODs and swimming.

### Behavioral functions of coordination of EOD production and tail movement

Our synchronized recordings of EODs and swimming revealed that EOD rate was correlated with swimming speed, with increases in EOD rate slightly preceding increases in swimming speed by approximately 40 ms (Fig. 1d). Although no objects or conspecifics were present in our recordings, this finding is consistent with previous studies conducted in the contexts of object approach (Hofmann et al. 2017), social interaction (Bell et al. 1974; Kramer and Bauer 1976), and prey capture (Arnegard and Carlson 2005), likely reflecting an increased need for sensory sampling. This temporal precedence of sensory sampling over movement parallels findings in other active sensing systems: in echolocating bats, changes in the emission angle of ultrasonic pulses precede flight turning (Ghose and Moss 2006), and in humans, changes in gaze direction precede changes in walking direction (Hollands et al. 2002). In line with these examples, our results support the idea that mormyrid electric fish likewise use sampled electrosensory information to guide swimming movements in darkness.

Detailed quantification of fish postures further revealed that increases in EOD rate preceded tail bending by approximately 130 ms (Fig. 1e). Active tail bending in mormyrid fishes has been described as one of the probing motor acts (PMAs), which are stereotyped behaviors composed of a series of swimming movements near an object (Toerring and Belbenoit 1979; Toerring and Moller 1984; von der Emde 1992). Our findings further suggest that tail movement can be temporally coupled to a brief EOD burst, or “scallop” (Fig. 1f) (Serrier and Moller 1989; Carlson 2002b; Carlson and Hopkins 2004b). Notably, a similar form of temporal coupling was found in echolocating bats, in which rapid ear movements are precisely coordinated with ultrasonic pulse–echo timing to actively modulate incoming acoustic signals (Yin and Müller 2019). Because mormyrid fish possess an electric organ in the tail and electroreceptors distributed over the body surface, tail movements have a strong impact on the encoding of electrosensory information during active sensing (Bastian 1999; Engelmann et al. 2008; Sawtell and Williams 2008; Sim and Kim 2011; Hofmann et al. 2013).

Mormyromast electroreceptors encode local EOD amplitude information, which is conveyed by their afferents to the hindbrain electrosensory lateral line lobe (ELL) (Szabo and Hagiwara 1967; Bell 1990; Perks and Sawtell 2022). The ELL forms a cerebellum-like circuit and receives a corollary discharge signal originating from the EOD command nucleus (CN), enabling the filtering of predictable electrosensory feedback (Bell et al. 2008; Requarth and Sawtell 2011; Fukutomi and Carlson 2020). A seminal series of studies further demonstrated that the ELL also receives movement-related signals, including swimming corollary discharge and proprioceptive feedback, allowing it to cancel electrosensory modulations associated with tail movements (Sawtell and Williams 2008; Sawtell 2010; Requarth and Sawtell 2014; Wallach and Sawtell 2023). These studies largely treated tail movements as a source of noise; however, the tail movements may also be used to efficiently exploit electrosensory information by generating structured sensory flow patterns (Hofmann et al. 2014, 2017). The neural mechanisms underlying the analysis of such sensory flow patterns remain to be addressed in future work.

PMAs have been associated with high-frequency EOD rates or sustained, regularized EOD rhythms in mormyrid fishes (Toerring and Belbenoit 1979; Toerring and Moller 1984; von der Emde 1992), while the scallop-like EOD pattern during PMAs was not reported to our knowledge. This discrepancy may arise from differences in task context (i.e., the presence or absence of an attractive object) and/or species-specific differences. The scallop EOD pattern has been documented in *B. niger* and *Brienomyrus brachyistius*, but not in other mormyrid species including *Gnathonemus petersii* and *Mormyrus rume* (Serrier and Moller 1989; Carlson 2002b; Carlson and Hopkins 2004b).

The EODs of mormyrid fishes have highly stereotyped waveforms that convey sender identity, including species, sex, and social dominance (Hopkins 1981; Bass and Hopkins 1983; Carlson et al. 2000; Feulner et al. 2009), and the amplitude itself is constant (von der Emde 1992). Because the electric organ acts as a dipole source, tail bending changes the orientation and spatial profile of the emitted electric field, thereby modulating the EOD amplitude received by neighboring fish. Previous studies have emphasized the importance of EOD intervals for electrocommunication (Moller 1970; Bell et al. 1974; Carlson 2002b), as well as the knollenorgan (KO) pathway specialized for processing temporal information (Bell and Grant 1989; Carlson 2009; Baker et al. 2013; Baker et al. 2016). In contrast, the functional significance of such amplitude-modulated (AM) components remains largely unexplored. KO responses to EODs are largely amplitude invariant except at low stimulus intensities, where spike probability decreases and response latency becomes more variable (Lyons-Warren et al. 2012). Because KO receptors are distributed across the body surface and the amplitude of the stimulus received by each receptor varies with the relative distance and orientation of the signaling fish, electric signaling coupled with tail movements can generate rich spatiotemporal patterns of KO population activity. Notably, some electrosensory midbrain neurons in this pathway are tuned to a specific range of stimulus amplitudes (Amagai 1998), suggesting a neural capacity to analyze AM components generated by coordinated EOD production and body movements.

### The optic tectum modulates both swimming and electric signaling behaviors

Our systematic microstimulation experiments showed that activation of OT neurons evokes both tail movements and increases in EOD rate (Fig. 2–3). Across all fish examined, EOD responses to OT stimulation occurred earlier than swimming responses, although the relative timing varied substantially with body size (Fig. 4c). This temporal order is consistent with our behavioral observations in freely swimming fish (Fig. 1). In contrast to fictive swimming, the latency of evoked EOD commands was negatively correlated with body length (Fig. 4b), contrary to our prediction that conduction delay should increase with body length. Possible explanations include compensatory mechanisms within the electromotor pathway, such as increased myelination or enlarged axon diameter (Grant et al. 1999).

Control of body-axis bending by the optic tectum has been reported across a wide range of vertebrates, from lampreys to salamanders, with output neurons in the deep layers projecting to reticulospinal neurons that command swimming movements (Herrero et al. 1998; Roeser and Baier 2003; Gahtan et al. 2005; Saitoh et al. 2007; Kardamakis et al. 2015; Helmbrecht et al. 2018; Flaive and Ryczko 2022). Consistent with this conserved circuit architecture, we found that stimulation at deeper sites of the optic tectum elicited more frequent swimming movements (Fig. 5), suggesting that a similar functional connection between the optic tectum (OT) and reticulospinal neurons is present in mormyrid fish (Requarth and Sawtell 2014). Whereas motor-related outputs arise from the deep layers, the superficial layers of the OT receive visual input from the retina in a topographically organized manner (Basso and May 2017; Isa et al. 2021), and in mormyrid fish, electrosensory inputs from the lateral nucleus of the torus semicircularis also terminate in the superficial layer in a corresponding topographic arrangement, providing a neural substrate for the formation of a cross-modal sensory map (Zeymer et al. 2018). Previous work in goldfish has shown that stimulation of posterior sites in the OT evokes larger tail movements than stimulation of anterior sites, suggesting the presence of a motor map along the rostrocaudal axis of the OT (Herrero et al. 1998), reflecting a transformation of this sensory topography into graded motor outputs. In our data, several fish exhibited a similar swimming motor map; however, this pattern was not robust across the population (Fig. 5). Such difference may reflect a swimming mode unique to electric fish, which allows backward movement without executing a turning maneuver, a capability not generally observed in other teleost fishes (Lannoo and Lannoo 1993; Hofmann et al. 2014; Skeels et al. 2023).

To our knowledge, the present study is the first to demonstrate that stimulation of the optic tectum can increase the EOD rate, in conjunction with the induction of fictive swimming. A similar experiment was performed in a previous study using another mormyrid species, *Gnathonemus petersii* (Requarth and Sawtell 2014). That study investigated how corollary discharge associated with tail movements modulates electrosensory processing in the ELL and demonstrated that fictive swimming can be evoked not only by stimulation of the OT but also by stimulation of the mesencephalic locomotor region (MLR). Although no quantitative analysis was performed, exemplar figures of motor nerve activity in that paper suggest that both the frequency of fictive swimming and the EOD command covary with the intensity of MLR stimulation. However, that study provides no evidence for a direct relationship between OT stimulation and EOD commands.

Our results clearly demonstrate that sites effective at eliciting EODs are not uniformly distributed within the OT; rather, the hotspot was localized to the posteroventral portion of the deep layer (Fig. 5). This result is in good agreement with anatomical findings showing that the ventral portion of the deep layer of the OT projects strongly to the dorsal part of the ventroposterior nucleus (VPd), a key component of the electromotor network (Wullimann and Northcutt 1990; Carlson 2002a). However, the functional significance of this spatial bias remains unclear. Taken together with the swimming results, these findings raise the possibility that, when a salient object is detected behind the animal, mormyrid fish may engage in backward swimming accompanied by high-frequency EOD production, whereas goldfish orient toward the object by bending the body axis (Herrero et al. 1998).

By what mechanisms does the OT regulate EOD production? Given the extremely short latency of evoked EOD commands (~ 10–20 ms; Fig. 4), direct connections between the OT and nuclei of the electromotor network, namely VPd and the thalamic dorsal posterior nucleus (DP), likely play a critical role (Carlson 2002a), despite the OT’s widespread connections to other brain regions (Wullimann and Northcutt 1990).VPd neurons receive EOD corollary discharge input originating from the CN and provide inhibitory input to DP and the precommand nucleus (PCN); both DP and PCN, in turn, provide excitatory input to the CN (Carlson 2002a, 2003). Thus, this network constitutes a central pattern generator based at least partly on recurrent inhibition (Marder and Bucher 2001). Taken together with these findings, our results suggest that OT neurons may inhibit VPd neurons, thereby transiently disinhibiting DP and PCN by reducing VPd-mediated inhibition. In addition, the OT–DP connection may be excitatory and could facilitate increases in EOD rate (Carlson and Hopkins 2004a). Future studies will be needed to further investigate the functional connectivity between the OT and VPd, as well as between the OT and DP.

### Neural mechanisms underlying coordination of multiple motor outputs

The OT, or superior colliculus (SC) in mammals, mediates sensorimotor transformations that underlie orienting behaviors across vertebrate species (Allen et al. 2021). Orienting behaviors often involve multiple motor outputs that are highly species-specific, yet can be reproduced by microstimulation of the OT/SC. For example, in primates, microstimulation of the SC evokes not only saccade-like eye movements but also head movements and pupil dilation (Stryker and Schiller 1975; Cooper and McPeek 2021; Wang and Munoz 2021). In rodents, microstimulation can elicit coordinated movements of the eyes, head, pinnae, and vibrissae (McHaffie and Stein 1982). In bats, microstimulation can evoke ultrasound vocalizations in addition to movements of the head and pinnae (Valentine et al. 2002). Here, we show that microstimulation of the OT in mormyrid electric fish evoked both swimming movements and transient increases in EOD production, extending the role of the OT/SC in coordinating species-specific orienting outputs to an active electrosensory system.

Although a large body of evidence suggests that the OT/SC is involved in coordinating distinct motor outputs during orienting movements, our understanding of the circuit mechanisms underlying this coordination remains limited. One study suggests the common drive hypothesis, in which the same population of efferent neurons drives multiple motor circuits (Wang and Munoz 2021). This hypothesis is supported by a correlation between saccade magnitude and the extent of pupil dilation evoked by microstimulations of the intermediate layer of SC (SCi) in rhesus monkeys, as well as by anatomical evidence that SCi neurons projecting to pontine premotor centers for saccade control give off axon collaterals that terminate in the central mesencephalic reticular formation, which in turn projects to the Edinger–Westphal nucleus involved in pupil control (Grantyn and Grantyn 1982; Scudder et al. 1996; May et al. 2016; Wang and Munoz 2021). Our results reveal a similar correlation between EOD rate and swimming activity evoked by OT microstimulation (Fig. 6). However, there is currently no evidence that neurons projecting from the OT to the electromotor network give off axon collaterals that also target reticular formation nuclei involved in swimming control (Carlson 2002a). Alternatively, this correlation may be mediated by local interneuronal circuits within the OT.

Unlike the intermittent actions exemplified by saccades, the coordination between EOD production and swimming in freely behaving mormyrid fish also includes continuous components (Fig. 1). The neural basis of this sustained coordination was not examined in the present study. Recurrent signals associated with swimming and EOD generation may play an important role in maintaining this coordination, and the OT may serve as a locus for integrating these signals, thereby contributing to the ongoing coupling of swimming and EOD production. Information about ongoing swimming movements may reach the OT as sensory feedback via somatosensory and mechanosensory lateral line pathways and may also be internally conveyed via corollary discharge (Meek 1983; Ali et al. 2023). Electrosensory feedback also terminates in the superficial layers of the OT, and VPd and DP neurons in the electromotor network project directly to the deep layers (Carlson 2002a; Zeymer et al. 2018). In echolocating bats, the SC has been shown to serve as a key hub linking sensory processing of echoes to the motor output of vocalization (Kothari et al. 2018; Wohlgemuth et al. 2018; Allen et al. 2021). By analogy, the mormyrid OT may play a similarly critical role in multisensory processing and in coordinating multiple motor outputs. Our results establish a functional link between the OT and both EOD generation and swimming, providing a foundation for future studies on the role of the OT in active electrosensing.

## Figures and Tables

**Figure 1:**
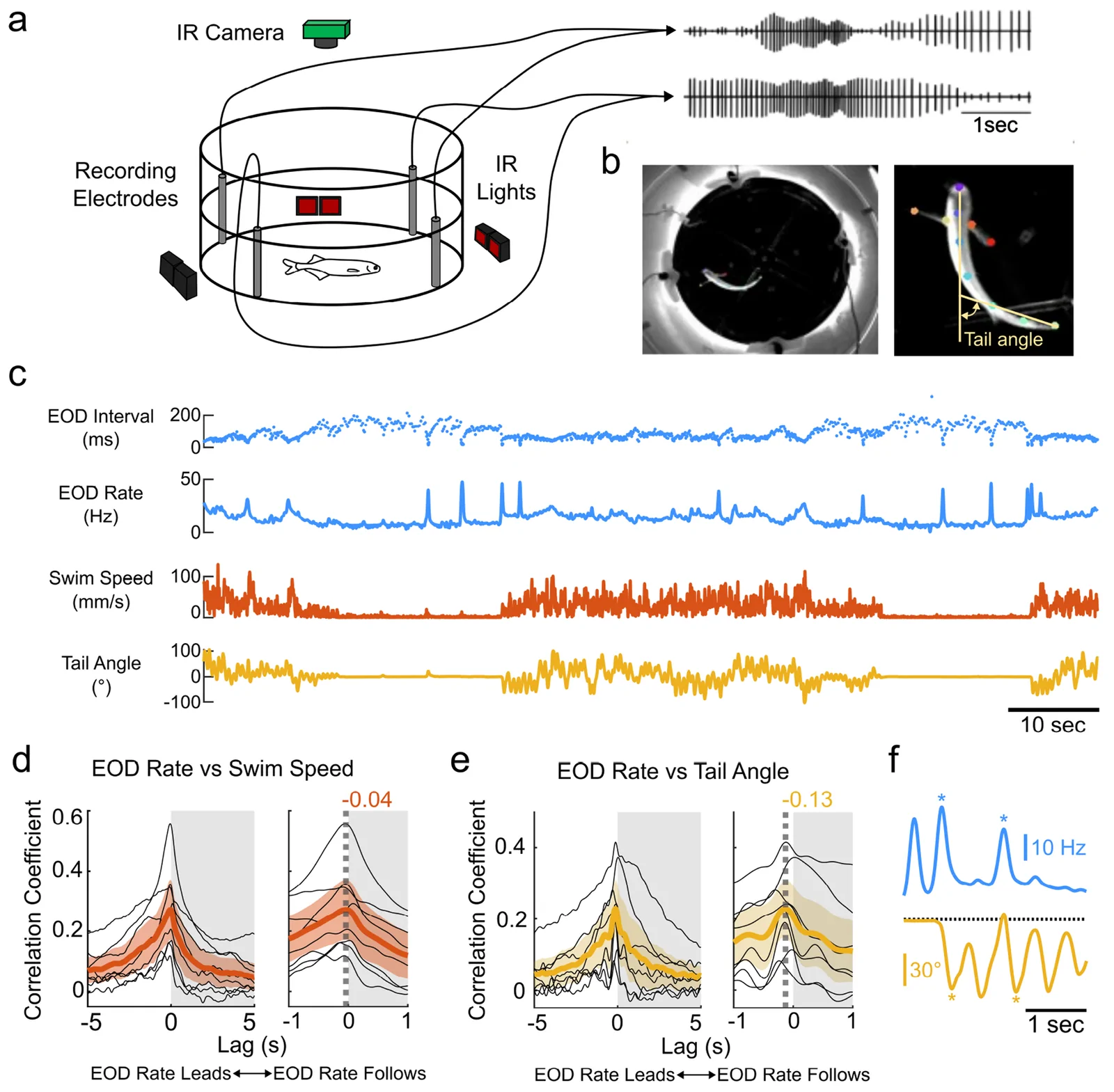
EOD rate correlates with swimming activity. (a) Experimental setup for simultaneous recording of electric organ discharges (EODs) and behavior from a freely swimming mormyrid fish in a circular tank. Swimming behavior was recorded in darkness using an infrared (IR)-sensitive camera positioned above the tank. Electric signals were recorded via two orthogonally arranged pairs of bipolar electrodes and amplified before digitization. (b) Pose tracking with DeepLabCut. A total of eleven body parts were tracked: seven along the body axis from snout to tail, plus the base and tip of each pectoral fin. Tail angle was defined as the angle between the first and third and the fifth and seventh body point axes. (c) Example traces of EOD interval, EOD rate, swimming speed, and tail angle. (d) Cross-correlation between EOD rate and swimming speed. Black traces show the cross-correlation coefficient for an individual fish as a function of time lag; a negative lag indicates EOD rate time series leads swimming speed, while a positive lag (gray region) indicates the opposite. The orange trace shows the average across all eight fish with shaded 95% confidence intervals. Right panel shows zoom-in of cross correlation with a vertical dotted line indicating the peak of the average trace. (e) The same as (d), instead showing the cross-correlation between EOD rate and absolute tail angle. (f) Zoomed-in example traces of EOD rate and tail angle from (c). Asterisks highlight time points where EOD bursts and tail bends are tightly temporally coupled.

**Figure 2:**
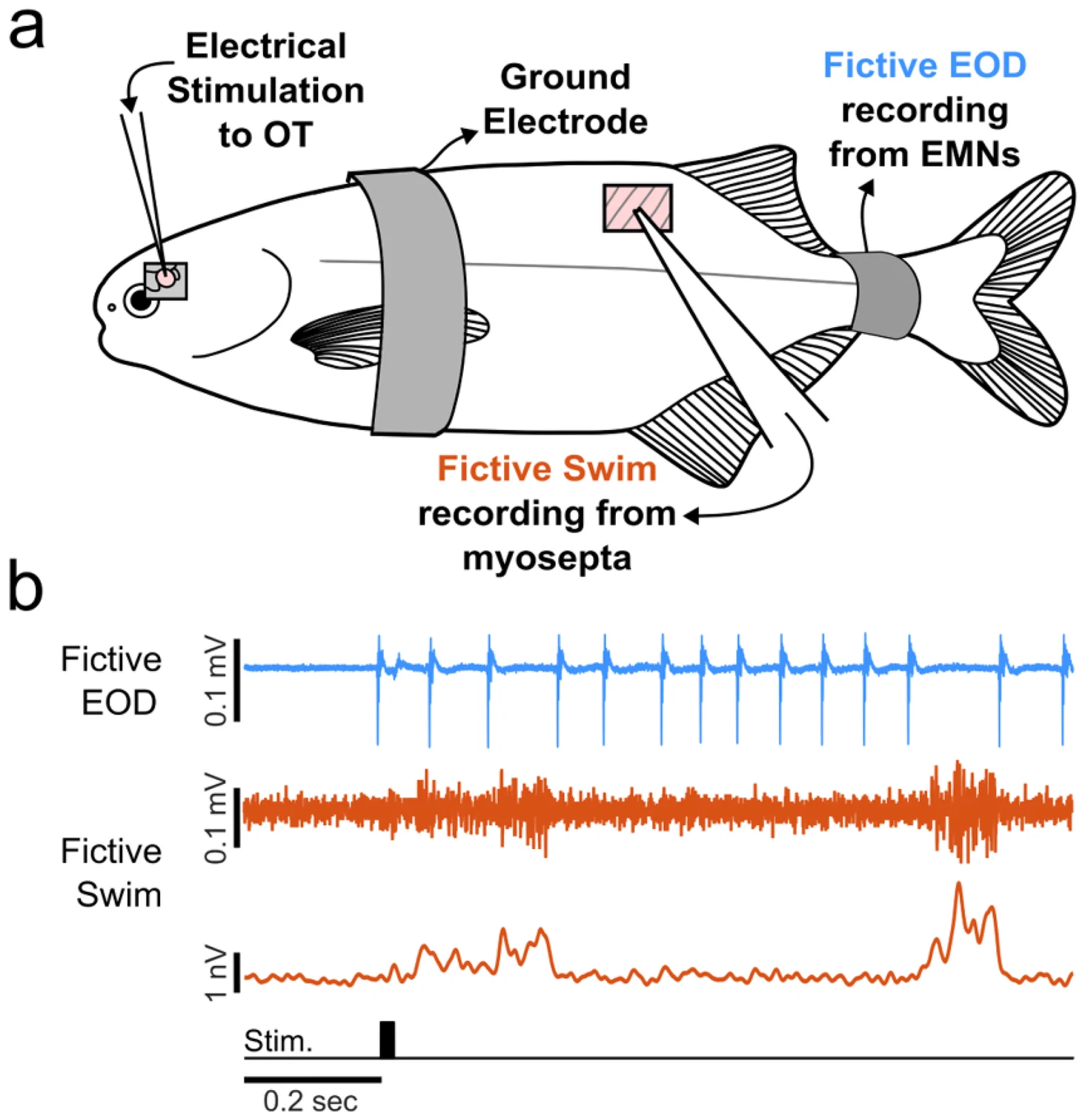
Electrical stimulation of the optic tectum evokes fictive EOD and fictive swim motor responses *in-vivo*. (a) Schematic representation of electrophysiological recording setup. Electrical stimulation is applied to the OT via concentric bipolar microelectrode. To record fictive swim motor responses, a suction electrode is attached to the myosepta between myomeres. Bipolar silver electrodes held in place by a plastic tube are placed around the tail to record fictive EOD responses from spinal electromotor neurons (EMN). A circular silver ground electrode surrounds the trunk of the fish. (b) Representative traces of fictive EOD and fictive swim obtained from the recording setup in (a) showing fictive EOD and fictive swim responses to electrical stimulation of the OT. Stimulation was applied as a 10-pulse train of 400 μs bipolar square pulses (100 μA, 100 Hz).

**Figure 3:**
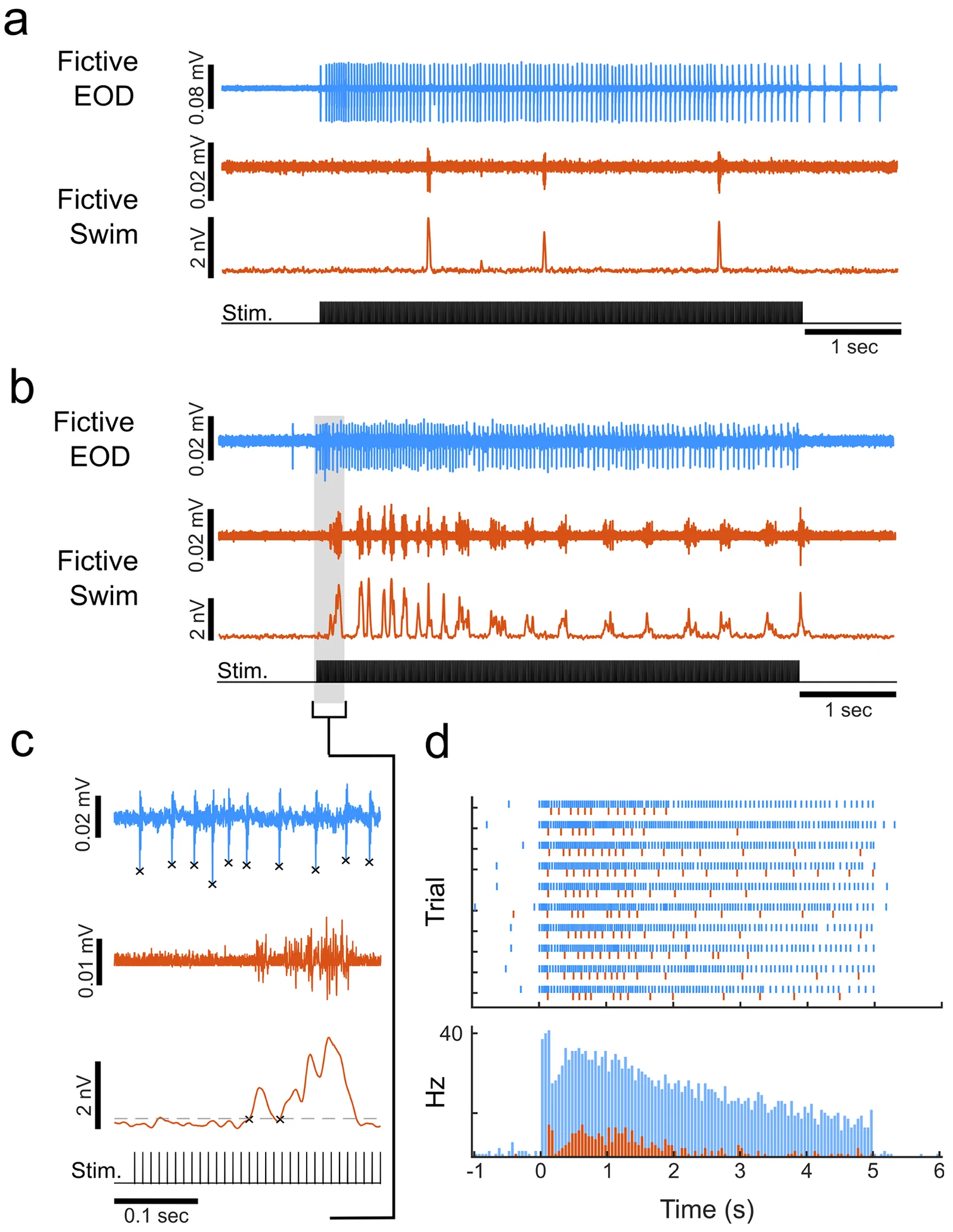
Extraction of fictive EOD and fictive swim timing. (a) Representative traces of fictive EOD and fictive swim obtained from the recording setup in Fig. 2a showing fictive EOD and fictive swim responses to electrical stimulation of the OT. Stimulation was applied for 5 seconds as a 500-pulse train of 400 ms bipolar square pulses (100 μA, 100 Hz). Example individual exhibits a high fictive swim response. (b) The same as (a), but for an individual who exhibits a low fictive swim response. (c) Zoom-in of (b); the onset timing of fictive EODs is defined as the first negative peak, represented by black ‘x’. Before determining the onset of fictive swim, raw traces are rectified and smoothed via Gaussian filtering (see Materials and Methods). The onset threshold (grey dashed line) was determined manually according to baseline noise levels for each individual. Onsets of fictive swim also represented by black ‘x’. (d) Raster plots show the onset timing of fictive EOD and fictive swim motor responses for a subset of 10 trials and the peri-stimulus time histogram (PSTH) for all 29 trials from the individual represented in (b) and (c) for a bin size of 50 ms.

**Figure 4:**
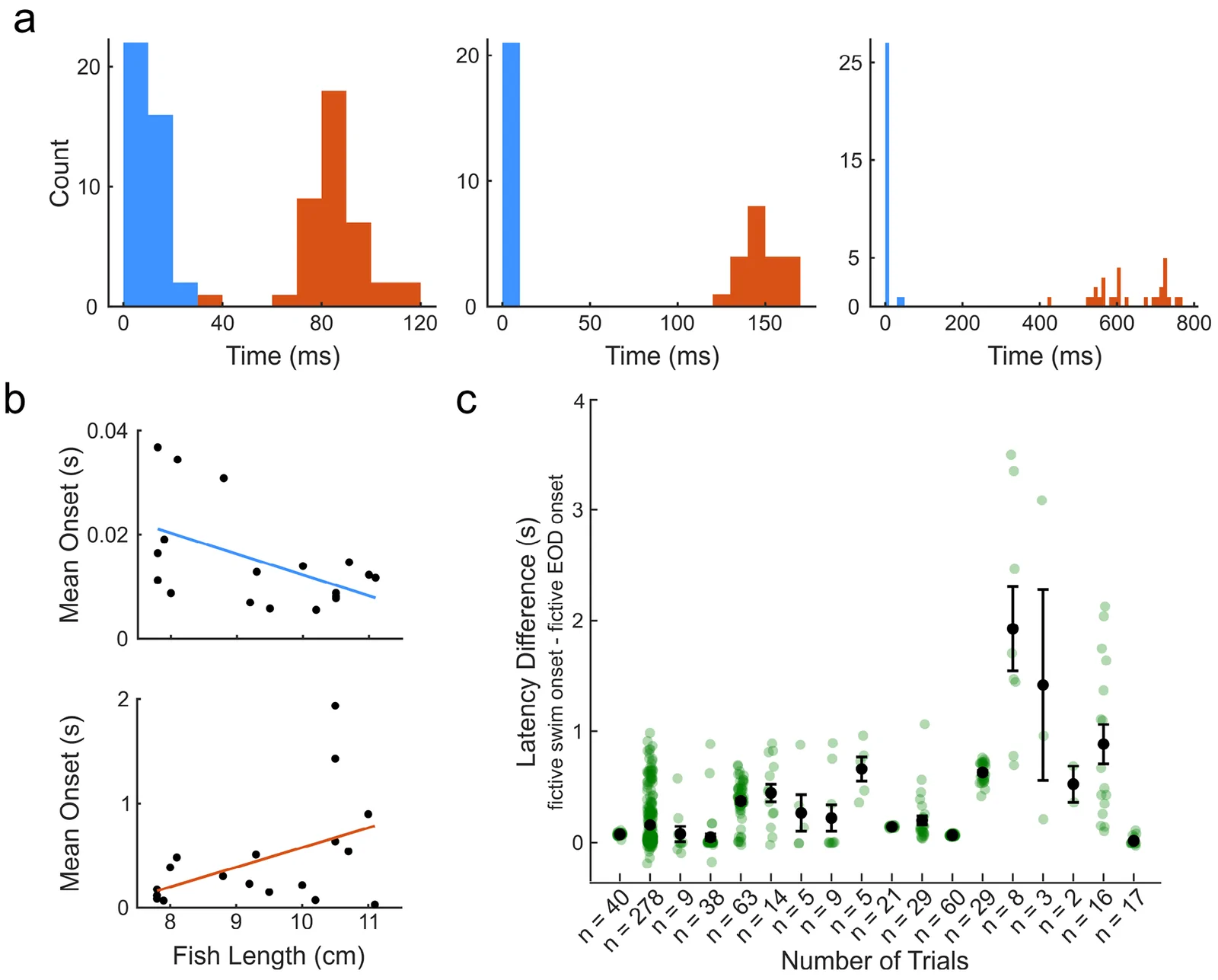
The delay between fictive motor responses shows a relationship with fish length. (a) Histograms showing when the onset of the first fictive EOD (blue) and fictive swim (orange) responses occur for three representative individuals to demonstrate individual variation. (b) Relationship between fish length and mean onset times for fictive EOD (top) and fictive swim (bottom). Regression lines were determined using linear regression. Slopes were −0.004 s/cm (fictive EOD) and 0.053 s/cm (fictive swim) and the intercepts were 0.053 s (fictive EOD) and −1.30 s (fictive swim). There is a significant negative correlation between fish length and average fictive EOD onset (R^2^ = 0.251, t_16_ = −2.31, *p* < 0.05) and a close to significant positive correlation between fish length and average fictive swim onset (R^2^ = 0.206, t_16_ = 2.04, *p* = 0.059). (c) The delay in seconds from the onset of the first fictive EOD to the onset of the first fictive swim for 18 individuals who exhibit distinctive bursts of fictive swim. Each point represents a single trial and error bars represent standard error of the mean. Individuals are organized from left to right according to increasing fish length. There is a near-significant positive correlation between body length and mean latency difference across individuals (R^2^ = 0.213, t_16_ = 2.08, *p* = 0.054).

**Figure 5:**
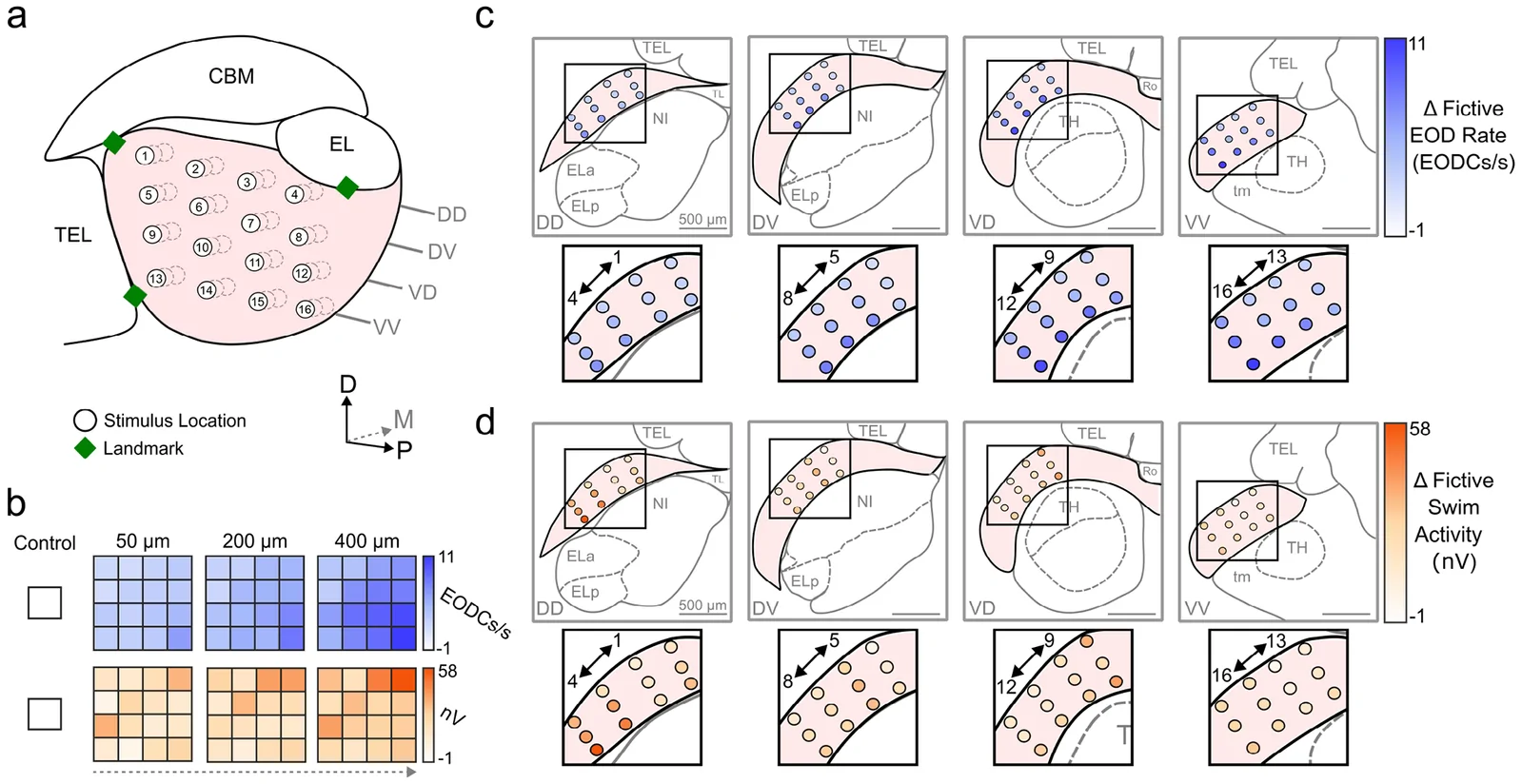
OT stimulation reveals spatial organization of fictive motor responses. (a) Schematic representation of OT stimulus locations. Landmarks for the calculation of the OT surface map are represented in green. Sites are numbered 1-16, represented by white circles, and stimulus depths are represented by dashed circles. Labels DD, DV, VD, and VV represent the relative locations of each row of stimulus sites. (b) Average heatmaps of fictive motor responses for 17 individuals. Columns from left to right proceed through the OT starting with the response to a control stimulus applied above the OT surface. The top row represents the change in rate fictive EOD production (EODCs/s) and the bottom row represents the change in fictive swim activity (nV). Colors progress from light to dark with the magnitude of change in fictive responses. Repeated measures ANOVA shows significant relationships between OT depth and fictive EOD rate (F_1, 33_ = 41.0, *p* < 0.001), as well as between OT depth and fictive swim activity (F_1, 33_ = 14.2, *p* < 0.001). Fictive EOD rate also depends on the dorsoventral axis (F_1, 50_ = 8.5, *p* < 0.01) and the anteroposterior axis (F_1, 50_ = 21.3, *p* < 0.001) whereas the fictive swim activity depends on neither the dorsoventral axis (F_1, 50_ = 1.2, *p* = 0.288) nor the anteroposterior axis (F_1, 50_ = 3.0, *p* = 0.091). (c) Schematic representations of relative anatomical locations of rows of stimulus sites shown in (a). Insets show stimulus sites with heatmap colors shown in (b) for fictive EOD responses. (d) Same as (c) but for fictive swim responses. Abbreviations: CBM, cerebellum; TEL, telencephalon; EL, exterolateral nucleus; ELa, anterior exterolateral nucleus; ELp, posterior exterolateral nucleus; TL, torus longitudinalis; NI, nucleus isthmi; Ro, nucleus rotundus; TH, thalamus; tm, tectum mesencephali.

**Figure 6:**
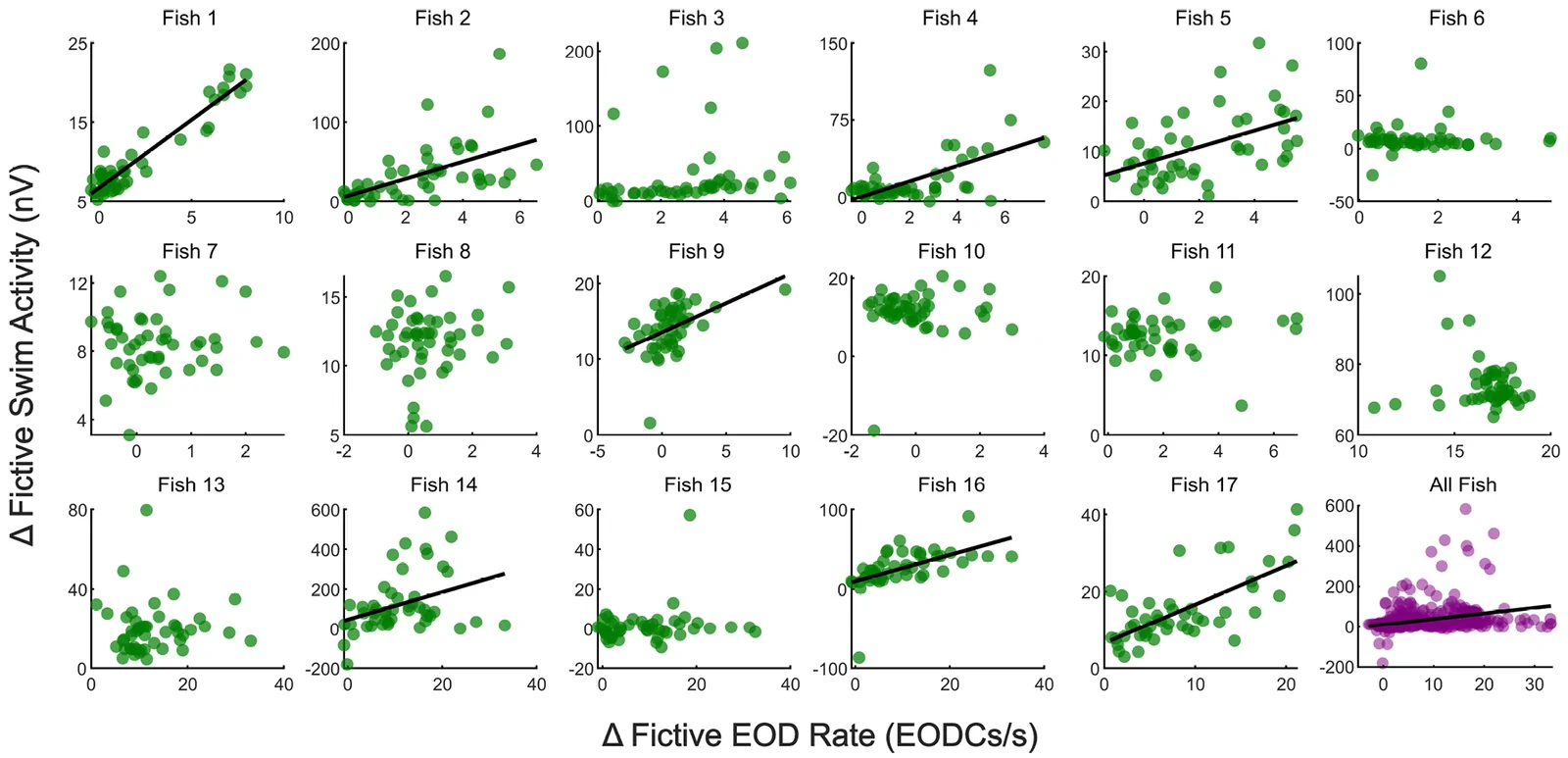
OT activation correlates with fictive EOD rate and fictive swim activity. Scatter plots for each of the 17 individuals from OT mapping experiments. Each green plot includes the values from all OT stimulation sites showing the relationship between the change in rate of fictive EOD production (EODCs/s) against the change in rate of fictive swim activity (nV). R^2^ values for all 17 fish range from 0.0000745 to 0.943. Linear regression shows significant slopes for 8 out of 17 fish (*p* < 0.05). Generalized linear mixed model (GLMM) was performed to confirm the relationship holds when controlling for individual variation, with fictive EOD rate as a predictor of fictive swim activity (β = 2.22 ± 0.317, *p* < 0.001). The purple plot includes all points from all 17 fish with the overall linear regression (R^2^ = 0.137, t_810_ = 11.3, *p* < 0.0001).

## Data Availability

The datasets and original codes are available from the corresponding authors upon request.
